# Advancements in dental bioreactor design: A comprehensive approach for application in dentistry

**DOI:** 10.1016/j.mex.2024.103026

**Published:** 2024-11-05

**Authors:** Esteban Astudillo-Ortiz, Pedro S. Babo, Ana I. Gonçalves, Manuela E. Gomes

**Affiliations:** a 3B's Research Group, I3Bs – Research Institute on Biomaterials, Biodegradables and Biomimetics, University of Minho, Headquarters of the European Institute of Excellence on Tissue Engineering and Regenerative Medicine, AvePark, Parque de Ciência e Tecnologia, Zona Industrial da Gandra, Guimarães 4805-017, Barco, Portugal; b ICVS/3 B's – PT Government Associate Laboratory, Guimarães 4805-017, Braga, Portugal; c Department of Endodontics, School of Dentistry, San Francisco de Quito University, Pampite and Diego de Robles, Quito 170901, Ecuador

**Keywords:** Bioreactors, Dental research, Endodontic tissue regeneration, Flow and Pressure-Controlled Bioreactor for Dental Vitality Maintenance and Dental Pulp Regeneration using Tissue Engineering Strategies

## Abstract

The protocol introduces a novel multi-chamber bioreactor tailored for ex-vivo cell culture in dentistry research, emulating the 3D dental environment to propel research in dental applications. Constructed primarily from a polymeric material with a sophisticated 3D design, the bioreactor securely holds teeth structures within sealed chambers, enabling controlled perfusion of culture medium crucial for cell growth through a singular entry and exit point. An integrated electronic system manages flow and pressure, ensuring precise control over environmental conditions. This technology facilitates cell cultivation under conditions closely resembling natural tooth microenvironments, offering opportunities for varied studies from understanding cellular behavior in dental contexts to targeted therapy development. The bioreactor's amalgamation of polymeric components, 3D design, and electronic controls enhances adaptability and accuracy, rendering it a valuable asset in dental research. The report comprehensively delineates the bioreactor's design, operations, and potential applications, showcasing its significant contributions to dental research and regenerative medicine. By amalgamating advanced technologies, this bioreactor emerges as a pivotal tool for investigating cellular processes within dental structures, paving the way for scientific exploration and therapeutic advancements.

## Graphical abstract

**Figure:**
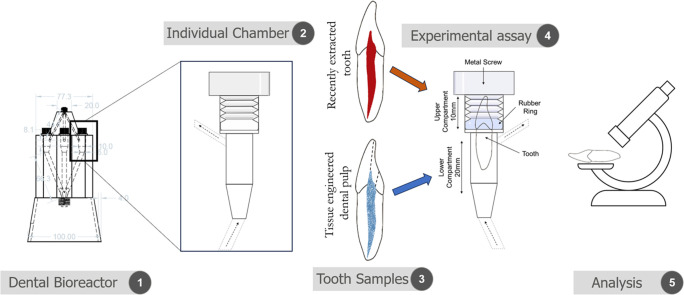

Specifications table

**Table:** 

Subject area:	Biological Sciences
More specific subject area:	Dental Pulp Tissue Engineering
Name of your method:	Flow and Pressure-Controlled Bioreactor for Dental Vitality Maintenance and Dental Pulp Regeneration using Tissue Engineering Strategies
Name and reference of original method:	Dynamic Hydrostatic Pressure Promotes Differentiation of Human Dental Pulp Stem Cells [1].
Resource availability:	https://bc-robotics.com/tutorials/controlling-a-solenoid-valve-with-arduino/ https://www.researchgate.net/publication/289860451_Control_system_for_continuous_positive_airway_pressure

## Background

Many efforts have been made to translate the extensive in vitro research into the clinical practice in endodontics, demonstrating promising outcomes [[1], [2], [3], [4], [5]]. However, the inherent limitations of clinical research studies, influenced by ethical considerations and operational costs, underscore the need for thorough histological and physiological validation of clinical outcomes [3]. Consequently, the pre-clinical assessment of tissue-engineered grafts in validated in vitro models becomes essential, anticipating the quality of regenerated tissues.

Translating newly developed endodontic biomaterials to clinical use encounters various challenges, including lack of appropriate in vitro models that can provide significative information to allow further stages leading to the transition of bench-side developed biomaterials to clinical applications [6]. The limitations of in-vitro assays to emulate the tissue response and the complex physiologic processes are difficult to overcome, [[7], [8], [9]], as for example the static nature of traditional in vitro culture environments where insufficient nutrient and oxygen flow through three-dimensional (3D) scaffolds leads to central cell necrosis in constructs [10]. To overcome this limitation, several studies exploring bioreactors applications have been published [11,12] that offer the possibility to establish dynamic environmental conditions for cells within tissue-engineered 3D scaffolds, thereby addressing the limitations of static culture [13]. Despite these advancements, disparities persist between in vitro and in vivo assays, demanding a specific focus on essential aspects such as blood flow and pressure, particularly in some applications.

Despite the irrefutable value of in-vitro assays, as mentioned above, the traditional static methodologies exhibit limitations in accurately recapitulating tissue responses and complex physiological processes [7,9]. In addition to the inefficient nutrient and oxygen flow typical of static culturing, bioreactors [[11], [12], [13]], strive to provide an environment that authentically mimics the dynamic conditions of living tissues.

In addressing these shortcomings, our proposal involves refining in-vitro methodologies and introducing a dental biomimetic bioreactor for enhanced screening before in-vivo experimentation. This strategic approach aims to bridge the gap between in-vitro and in-vivo results, ensuring a more accurate representation of biomaterials and tissue engineering strategies in a clinical context. By focusing on early evaluation in regenerative endodontics, we strive to improve the translational success of biomaterials from bench-side development to practical clinical applications. Consequently, this technical report highlights advancements in a perfusion multi-chamber bioreactor designed for ex-vivo cell culture, featuring electronic flow and pressure control. Specifically tailored for endodontic tissue engineering, the bioreactor aims to simulate a dynamic tissue environment. One of the mail goals of the proposed system consists in maintaining the vitality of extracted teeth samples for biomaterial assessment studies. Another major goal of the proposed bioreactor is to replicate the dental pulp tissue microenvironment for in-depth research in tissue engineering approaches targeting dental applications. The report details the bioreactor's design, functionality, and potential applications. Furthermore, proof of concept experiments were performed that highlight its dual role in preserving teeth for ongoing dental biomaterial research and for facilitating assessment of dental pulp tissue engineering approaches.

## Method details

### Dental bioreactor characteristics

#### Bioreactor design

**Fig. 1:**
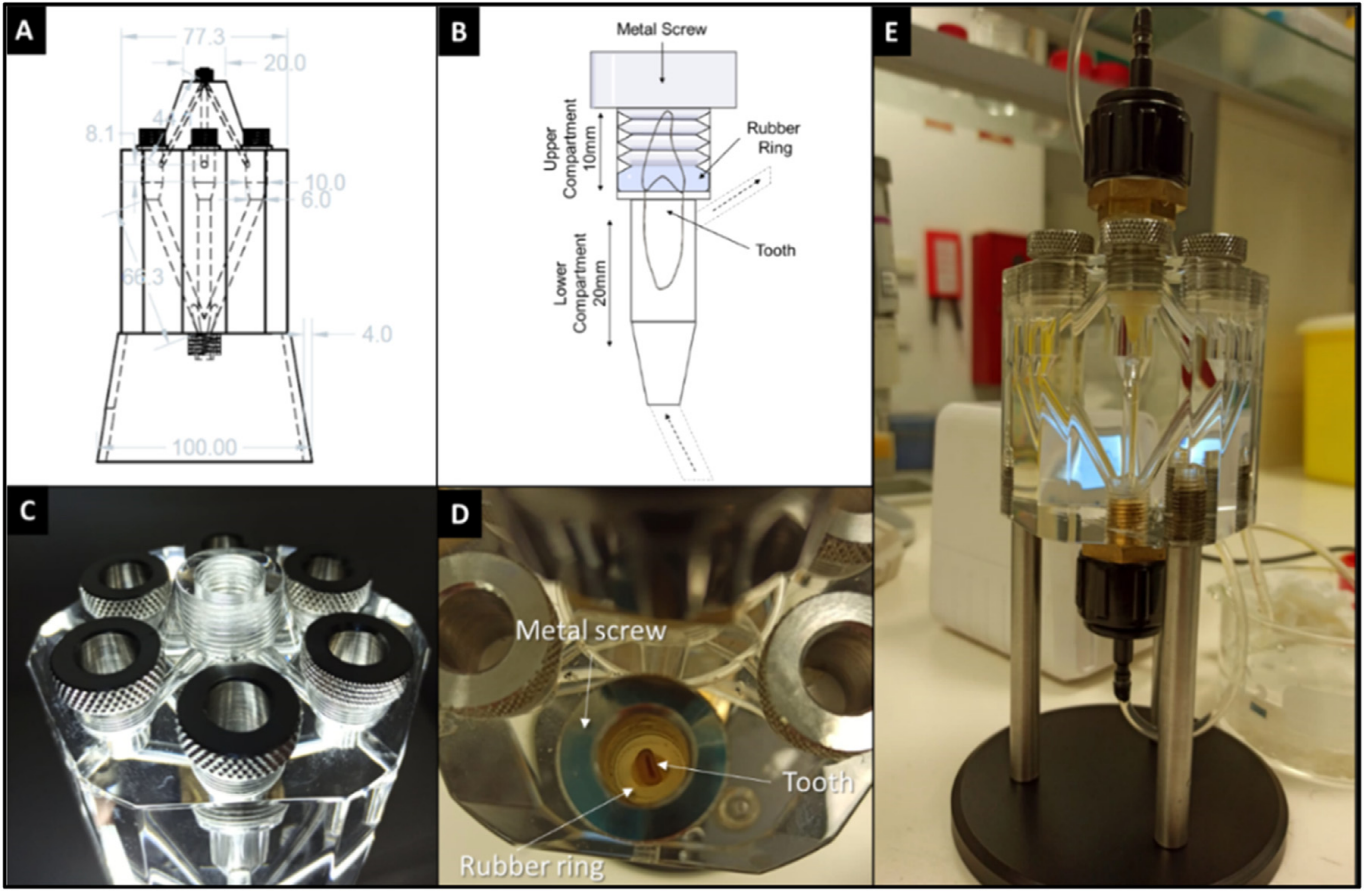
Bioreactor design. A. The bioreactor draft designed in Autodesk Autocad 2019 student version. This visual representation offers a comprehensive view of the dimensions of each constituent element, providing details about the design and structure of the bioreactor draft. B. Individual chamber features the tooth-holding system designed to anchor the tooth securely within the chamber. It also emphasizes the chamber's capability to effectively isolate distinct compartments. C. Actual photograph showcasing the dental bioreactor, highlighting its six chambers, each comprised of two distinct compartments. D. Upper compartment showing a tooth being anchored by the tooth-holding system. E. Dental bioreactor being connected to the flow system.

The bioreactor design, created using Autodesk Autocad 2019 student version, features a polymeric main part with hexagonal format, made of polycarbonate. Six cylindrical chambers (30mm high, 10 mm diameter) were milled inside the main part, each equipped with two compartments. The upper compartment (10mm high) includes a threaded section for a hollow metal screw. This screw functions to compress a rubber ring, enabling to secure the tooth and simultaneously establishing a hermetic seal for the chamber. In the lower compartment, the tooth root is exposed to a hermetically sealed chamber, where a circulating culture medium mimics the blood flow and pressure of the dental pulp (Fig. 1).

#### Integrated electronic system to control flow and pressure manufacturing

**Fig. 2:**
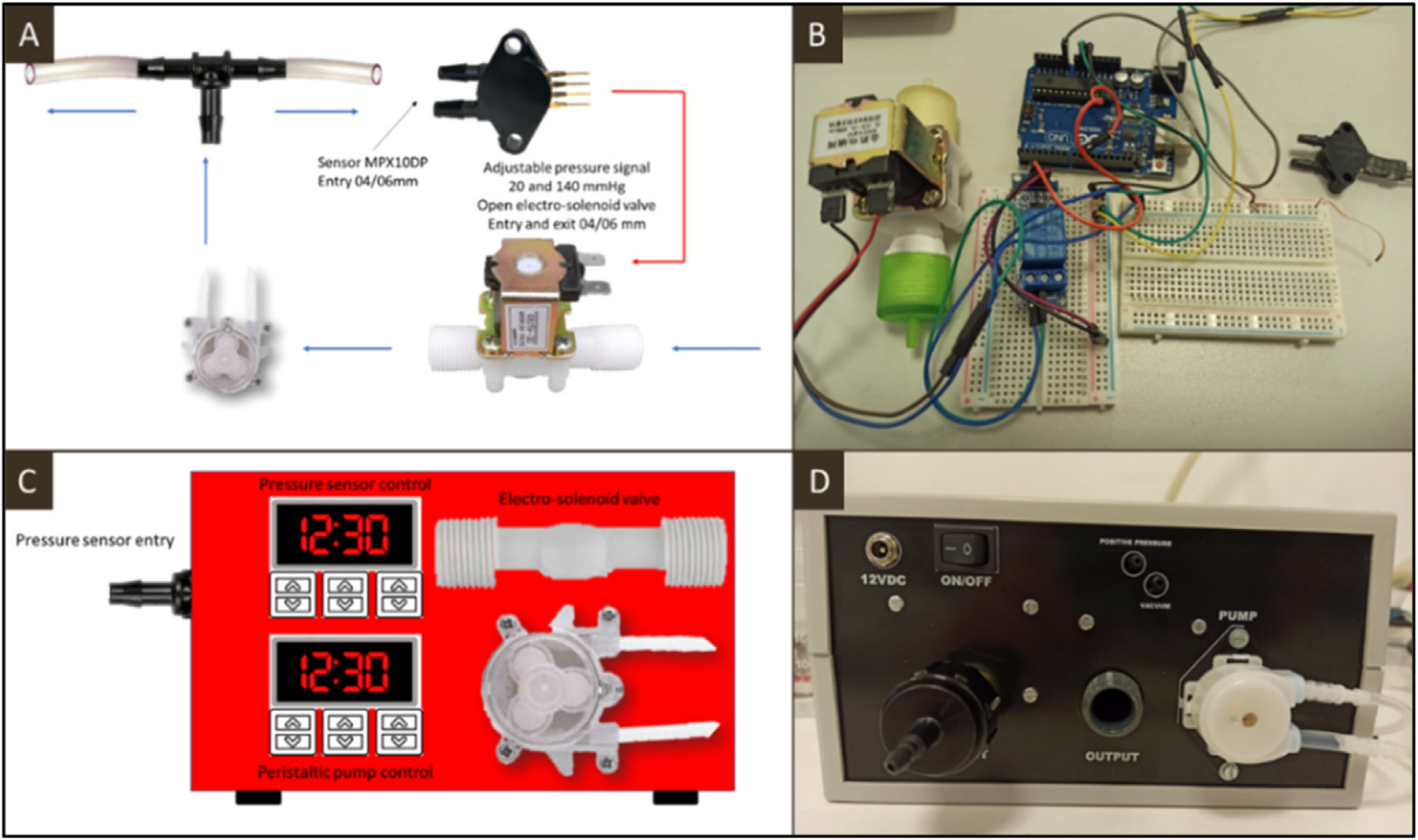
Design of the integrated electronic system to control flow and pressure. A. Schematic design of the flow and pressure control system. B. Actual photography of the flow and pressure control system. It includes a solenoid valve connected to a pressure sensor through a relay module mounted on an Arduino Uno plaque. C and D. Schematic and actual flow and pressure control system apparatus design.

The dental bioreactor incorporates flow and pressure control mechanisms designed to closely emulate the natural environment of the dental pulp. This control system, developed using Arduino technology, integrates a pressure sensor (MPX10DP, unbranded), solenoid valve (12 V DC, outer diameter 1/2″, unbranded), and peristaltic pump (12 V DC IntlLab). These components synchronize the flow and pressure, ensuring precise and optimal conditions within the dental bioreactor (Fig. 2).

### Configuration of the flow and pressure control system

**Fig. 3:**
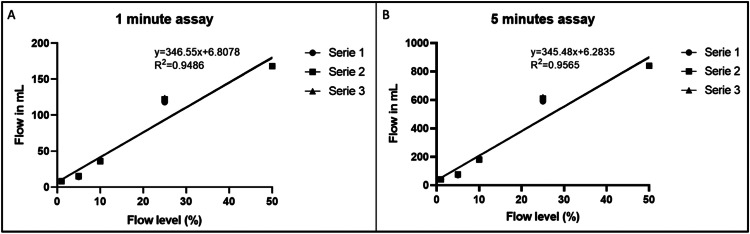
Coefficient determination analysis for optimizing control flow accuracy. A. The assay was conducted under a constant water flow for 1 minute, and B. The analysis was extended to 5 minutes.

The peristaltic pump's flow was measured using a consistent water flow for 1 and 5 minutes across five different levels (1%, 5%, 10%, 25%, and 50%) with 3 replicas (Table 1, Table 2). The coefficient of determination (R²) analysis resulted in a value of 0.9488, demonstrating the pump's robust predictive capacity and ability to achieve precise flow control (Fig. 3).

**Table 1:** Peristaltic pump's flow measurement during 1 minute.

Percentage	1	2	3	Average	SD
1%	8.2	8.2	8.2	**8.2**	0.00
5%	14.2	15.2	15.2	**14.9**	0.58
10%	37.4	36	37.6	**37**	0.87
25%	118	122	124	**121.3**	3.06
50%	168	168	168	**168**	0.00

**Table 2:** Peristaltic pump's flow measurement during 5 minutes.

Percentage	1	2	3	Average	SD
1%	41	41	41	41	0.00
5%	71	76	76	74.3	2.89
10%	187	180	188	185	4.36
25%	590	610	620	606.7	15.28
50%	840	840	840	840	0.00

In addition, the pressure valve was adjusted to 28 mmHg, according to the actual pulp pressure [14], corresponding to a pressure sensor MPX10DP reading of 144 mV. The programmed command dictates that the relay deactivates when the pressure exceeds 144 mV, closing the solenoid valve. Conversely, if the pressure falls below 144 mV, the relay activates, enabling liquid flow until reaching the specified value, thereby sustaining a constant pressure at 28 mmHg. This setup mimics a pulsatile liquid pumping reminiscent of physiological pulses observed in living organisms [15].

Regarding the flow, configuring pulp pressure proved challenging due to anatomical variability in the specimens. To overcome this, the flow was adjusted in channels connecting bioreactor chambers, simulating the mandibular artery flow [16]. The calculations utilized the diameter and flow range (0.8–1.8 mm; 0.7–3.7mL/min), tailored to match the diameter of the bioreactor inter-chamber channels (6mm) as depicted in Fig. 1A. This adaptation addressed anatomical variations, ensuring a meaningful simulation in the bioreactor setup. Considering the 6 mm diameter of the inter-chamber channels, flow rates of 12.33 and 23.33 mL/mm were identified as essential. Consequently, a 5% flow level was chosen for the study (Table 1).

### Proof of concept experiment 1 - Extracted tooth vitality maintenance assay

#### Tooth installation in the bioreactor

Two mandibular premolars preserved in a falcon tube with physiological saline solution, were transported to the laboratory in about 30 minutes upon extraction. Subsequently, the teeth underwent a cleansing procedure involving washing with milli-Q water and scrubbing with sterile gauze embedded in tetracycline. One tooth was placed in one of the individual chambers of the bioreactor and secured using a tooth-holding system, while Dulbecco's Modified Eagle's Medium-low glucose (DMEM, Sigma-Aldrich, St. Louis, USA) supplemented with 10% fetal bovine serum (FBS, ThermoFisher Scientific, Waltham, USA) and 1% A/A circulated through the systems. This method was termed dynamic culture. The medium DMEM is a widely used basal medium for supporting the growth of many different mammalian cells. The standard 5% CO_2_ environmental parameter mimics physiological conditions typically used in tissue culture, and it also indirectly regulates the pH of the culture medium via the bicarbonate buffer of the medium, to its physiological range. The medium was pumped at a 5% flow rate and a pressure of 28 mmHg, ensuring sufficient nutrient delivery and waste removal in the dynamic system. The other tooth, serving as a control, was fully immersed in a petri dish containing 10 mL of the same DMEM supplemented with 10% FBS and 1% A/A. This method was termed static culture. Both samples were cultured for 14 days at 37°C with 5% CO2, with medium changes twice a week.

### Proof of concept experiment 2 - Dental pulp regeneration assay using a tissue engineering strategy

#### Expansion of human dental tissue derived cells

Human dental pulp cells (DPCs) were extracted from human third molars following the methodology established in previous studies [17]. These cells were cultured in 75 cm^2^ tissue culture flasks using DMEM, supplemented with 10% FBS and 1% A/A. The culture medium was changed twice a week, and the cell cultures were maintained semi-confluent. All cultures were incubated at 37°C and 5% CO_2_. Cells within passage 4 were utilized for the experiments conducted in this study.

#### Tooth root preparation and filling

Six mandibular premolars stored in physiological saline were prepared using a 1.2 mm diameter cylindric diamond drill and subjected to Aksel's dentin conditioning protocol with some modifications [18]. The conditioning involved sodium hypochlorite, milli-Q water, EDTA, and PBS.

Subsequently, an aldehyde-hydrazide hyaluronic acid-based hydrogel enriched with platelet lysate was prepared in sterile conditions using established protocols by our group and described in previous publications [19,20]. Briefly, aldehyde-modified hyaluronic acid and hydrazide-modified hyaluronic acid were dissolved in PBS and platelet lysate at 2 wt%, respectively, followed by sterilization through UV irradiation before cell encapsulation.

After confluence, DPCs were subjected to trypsinization, followed by suspension in culture media, cell counting, and centrifugation at 300 xg for 5 minutes at 22°C. After discarding the supernatant medium, approximately 8 × 10^6^ cells per mL were reconstituted in a hydrazide-modified hyaluronic acid solution dissolved in platelet lysate. This mixture was thoroughly blended using a piston pipette in an up-and-down motion.

A sterilized double-barrel syringe (labeled A and B) was used to load the hydrogels, incorporating a static mixer at the outlet and a gauge needle attached to the tip. Barrel A was filled with hydrazide-modified hyaluronic acid, previously dissolved in platelet lysate, while barrel B contained the cell mixture. Each root canal was filled with a 10µL aliquot of the designated cell-hydrogel combination. Subsequently, the pulp chamber access of each tooth was hermetically sealed utilizing a restorative foundation comprising mineral trioxide aggregate (MTA; Cerkamed), capped with glass ionomer (Vitrebond 3 M). Finally, the six dental samples filled with the cell-laden hydrogel were placed in the bioreactor under identical conditions.

## Method validation

### Histological processing

After completing the designated culturing time points, both teeth were collected and thoroughly rinsed with PBS. Afterwards, they were immersed in a 10% (v/v) neutral buffered formalin solution (ThermoFisher Scientific, Waltham, USA) for 48 hours. Subsequently, a profound sagittal groove was incised into the teeth using a diamond disc, and a cement spatula was employed to execute a lever movement, facilitating the controlled fracture of the teeth into two halves. This method enabled the pulp extraction while preserving the integrity of the inner tissues. The fixed dental pulps underwent dehydration in a series of graded alcohol solutions. Subsequently, the processed specimens were embedded in paraffin wax to prepare them for histological and immunohistochemical analyses. Thin sections, measuring 4 µm in thickness, were then cut and subjected to routine staining with hematoxylin and eosin for subsequent microscopic analysis.

**Fig. 4:**
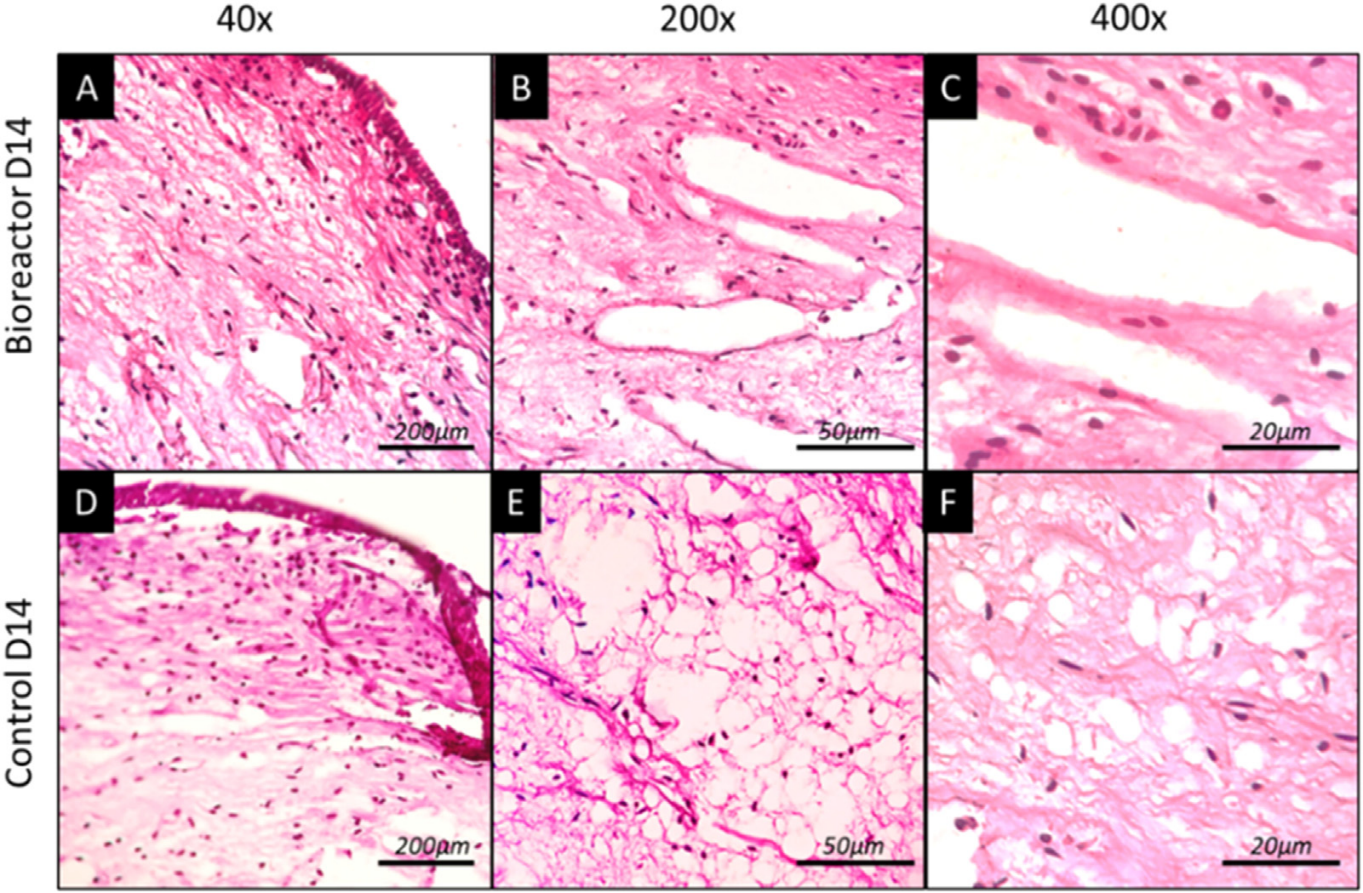
H&E analysis of extracted tooth in dynamic and static culture conditions. A. Micrograph depicting a dental pulp sample maintained for 14 days in dynamic culture conditions at 40x magnification. B. Micrograph illustrating the pulp tissue at 200x magnification, showcasing a cluster of blood vessels surrounded by loose connective tissues. C. Further magnification at 400x, providing a detailed view of the vascularized environment within the dental pulp. D. Micrograph presenting a dental pulp sample cultured for 14 days in static culture conditions at 40x magnification. E. Close-up view revealing an area of accumulated adipose-like tissue amidst the loose connective tissue at 200x magnification. F. Enhanced magnification at 400x, offering a closer examination of the adipose-like tissue within the context of the surrounding connective tissues.

Upon microscopic examination, tooth samples cultured under dynamic conditions revealed a uniform structure featuring aligned odontoblasts with basal nuclei notably situated in the superficial layer of the tissue (Fig. 4). The histological examination reveals distinct zones within the dental pulp, comprising a well-vascularized cell-free zone, a fibrillar cell-rich zone, and a characteristic pulp proper. Notably, the larger blood vessels exhibit a diminished population of endothelial cells within their lumens; however, perivascular cells maintain a normative expression. Additionally, there is a noteworthy augmentation of fibers and a diminution of the ground substance in the extracellular matrix (ECM) (Fig. 4).

By examining the control tooth cultured under static conditions, detachment of the odontoblastic layer from the cell-free zone was noted, along with observable intercellular spaces and nuclei that were not fully discernible. Notably, no distinct cell-free zone was observed. The cell-rich zone exhibited numerous thick fibers, no ground substance, and a lack of blood vessels. The dental pulp proper displayed pale staining, indicative of poorly vascularized tissue, with notable accumulations of adipose-like tissues (Fig. 4).

While the histological findings presented herein do not independently ascertain pulp tissues' vitality, they provide valuable insights. Specifically, under dynamic culture conditions employing controlled perfusion of culture media with regulated flow and pressure, the histological characteristics exhibited by pulp tissues resemble those observed in healthy dental pulp. This suggests that dynamic culture conditions may better maintain the histological integrity of pulp tissues than static culture conditions. However, further comprehensive investigations are warranted to elucidate these histological resemblances' functional implications and validate their significance in broader physiological contexts.

### Fluorescence microscopy

Following the dental pulp regeneration assay using a tissue engineering strategy, teeth were retrieved from the bioreactor and subjected to a PBS rinse. Subsequently, a protocol for cell fixation previously established by our research group was employed [20]. Briefly, the specimens underwent PBS washing before and after fixation in 10% (v/v) neutral buffered formalin for 30 min. Following fixation, teeth were immersed in a solution of 0.2% (v/v) Triton X-100 in PBS for 1 hour at room temperature withslight movements. Subsequently, the specimens were exposed to a PBS solution containing 4′,6-diamidino-2-phenylindole dilactate (DAPI, Biotium, San Francisco, USA) at a ratio of 1:1000 v/v and Phalloidin–Tetramethylrhodamine B isothiocyanate from Amanita phalloides (Sigma-Aldrich, St. Louis, USA) at a ratio of 1:200 v/v, for 1 hour at room temperature under mild agitation. The samples were washed thrice in PBS for five minutes each to minimize background fluorescence. Then, the specimens were observed using a Fluorescence Inverted Microscope (Zeiss, Axio Observer Z1, Göttingen, Germany), and representative micrographs were captured.

**Fig. 5:**
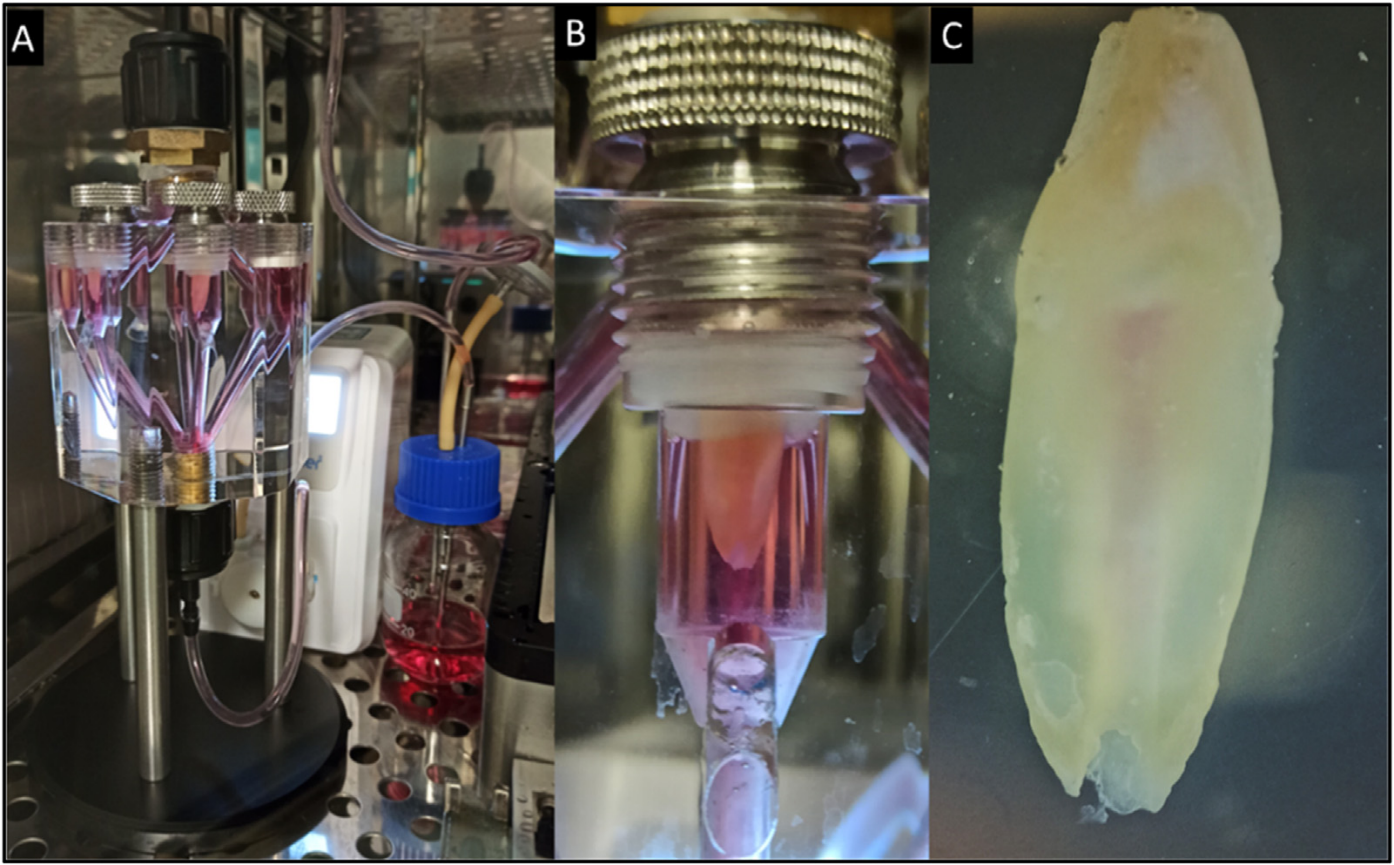
Dental Pulp regeneration assay. A. dental bioreactor operational within an incubator, ensuring controlled conditions of temperature, humidity, and CO_2_ levels. B. Individual chamber depicted, securing the tooth through the tooth-holding system, with the root canal visibly observed at a macroscopic level. C. Retrieved tooth after 14 days of dynamic culture.

Throughout the experiment, it was noted that the hydrogel-cell mixture consistently retained its macroscopic homogeneity within the prepared root canals over the 14-day assay period. Upon changing the culture medium and retrieving samples from the dental bioreactor, it became evident that the hydrogel had assimilated red phenol from the culture medium, resulting in discernible pigmentation (Fig. 5).

**Fig. 6:**
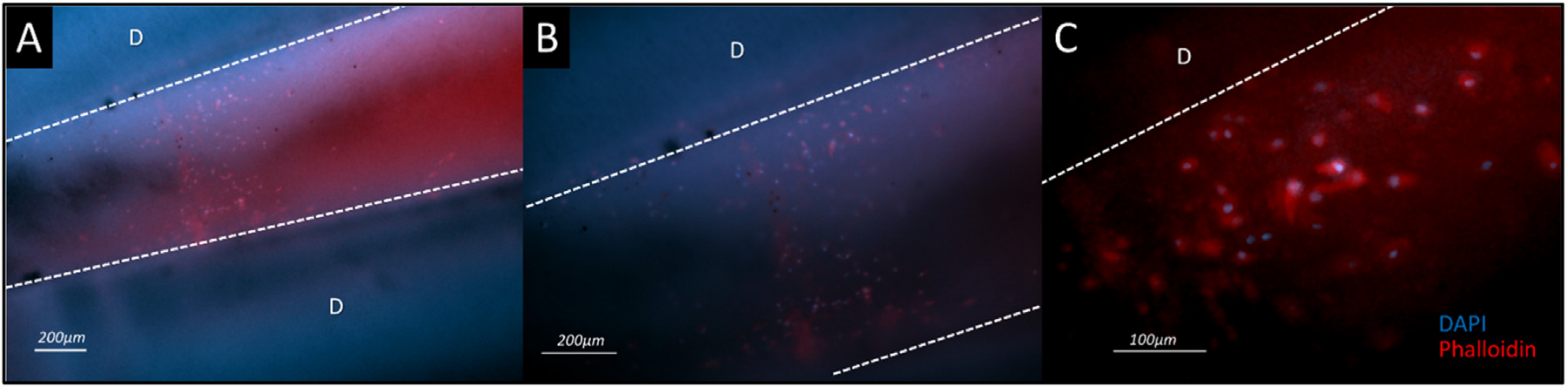
Dapi-Phalloidin staining after 14 days in dynamic culture. A. A wide view of the whole root canal depicting a cluster of cells encapsulated into the aldehyde-hydrazide hyaluronic acid-based hydrogel enriched with platelet lysate in a 4x augmentation. B. A magnified view of the cellular conglomerate region allows for detailed observation of cell morphology and arrangement at 10x magnification. C. A representative segment of the DPCs cluster visualized under a magnification of 40x.

Following the conclusion of the dynamic culture test, teeth retrieved from the dental bioreactor were halved, as previously described in the methodology, and the root canal contents underwent DAPI-phalloidin staining. This staining revealed the viability of dental pulp cells (DPCs) after 14 days in a dynamic culture (Fig. 6). Notably, the hydrogel exhibited a dense population of viable cells. The cells exhibited a fusiform morphology in the central region of the root canal. However, as the cells approached the dentin wall, their configuration transitioned to an oval and cubic shape.

Dapi-Phalloidin staining to assess cell vitality within our bioreactor. This method allowed us to visualize cell nuclei and cytoskeletal structures, revealing notable variations in cell morphology near the dentin walls. These observations confirm that cells remain viable and responsive, indicating the release of growth factors from the decellularized mineralized matrices, which mimic the dental pulp environment. Dapi-Phalloidin staining validates our protocol as effective for replicating the physiological conditions necessary for dental pulp tissue engineering by demonstrating the maintenance of cell vitality and the induction of cellular responses.

## Limitations

Within the constraints of the current investigation, pertaining to the initial objective, it was discerned that the pulp tissue preserved its inherent attributes considering the normal characteristics of a sound dental pulp reported by Galler in 2021 [21]. Slight alterations were noted in the vascular endothelial structure (Fig. 4). The phenomenon referred to as endothelium denudation is characterized by the removal or depletion of endothelial cells from the vessel walls. This occurrence is posited to be influenced by multiple factors, including the dynamic fluid shear stress induced by the flow rate of the culture medium over a duration of 14 days, the reduction in oxygen concentrations, and the lack of a culture medium tailored specifically for the maintenance of epithelial cells [[22], [23], [24], [25]].

## Ethics statements

Human teeth used in the present study were removed for orthodontic purposes at the Service of Oral Surgery of Malo Clinic Dental Care in Portugal, following established quality and safety standards (as established in 2004/23/CE, 2006/17/CE, and 2006/86/CE). The extraction procedures followed an institutional board protocol approved by the Ethical Commission for Health on 23/12/2014. All human donors provided written informed consent before enrollment, aligning with the Declaration of Helsinki (59th WMA General Assembly, Seoul, Korea, October 2008).

## Declaration of competing interest

The authors declare that they have no known competing financial interests or personal relationships that could have appeared to influence the work reported in this paper.

## Data Availability

Data will be made available on request.
